# An Engineered Version of Human PON2 Opens the Way to Understand the Role of Its Post-Translational Modifications in Modulating Catalytic Activity

**DOI:** 10.1371/journal.pone.0144579

**Published:** 2015-12-10

**Authors:** Luigi Mandrich, Mariangela Cerreta, Giuseppe Manco

**Affiliations:** Institute of Protein Biochemistry, National Research Council, Via Pietro Castellino 111, 80131 Naples, Italy; University Paris Diderot-Paris 7, FRANCE

## Abstract

The human paraoxonase 2 (PON2) has been described as a highly specific lactonase hydrolysing the quorum sensing molecule *N*-(3-oxododecanoyl)-L-homoserine lactone (3OC12-HSL) and having secondary esterase but not phosphotriesterase activity, in contrast with the related enzymes PON1 and PON3. It has been suggested that PON2 enzyme activity is dependent on glycosylation and its N-terminal region has been recently demonstrated to be a transmembrane domain mediating association to membranes. In the present study we describe a mutated form of PON2, lacking the above N-terminal region, which has been further stabilized by the insertion of six amino acidic substitutions. The engineered version, hence forth called rPON2, has been over-expressed in *E*.*coli*, refolded from inclusion bodies and purified, yielding an enzyme with the same characteristics as the full length enzyme. Therefore the first conclusion of this work was that the catalytic activity is independent from the N-terminus and protein glycosylation. The kinetic characterization confirmed the primary activity on 3OC12-HSL; accordingly, *in vitro* experiments of inhibition of the biofilm formed by *Pseudomonas aeruginosa* (PAO1) have demonstrated that rPON2 is more effective than PON1. In addition, we observed small but significant activity against organophosphorothiotes pesticides, m-parathion, coumaphos and malathion.The availability of fair amount of active protein allowed to pinpoint, by mass-spectrometry, ubiquitination of Lys 168 induced in rPON2 by HeLa extract and to correlate such post-translational modification to the modulation of catalytic activity. A mutational analysis of the modified residue confirmed the result.

## Introduction

In the last few decades there has been an increased interest in human paraoxonases (PONs), because a number of diseases has been related to these proteins [1, 2]. The family comprises three members: paraoxonase 1 (PON1), paraoxonase 2 (PON2), and paraoxonase 3 (PON3), encoded by three different genes located in a cluster of chromosome 7 [3]; these genes share about 70% sequence identity, suggesting their origin from a common precursor [4, 5]. PON1 and PON3 expression has been observed mainly in the liver and kidneys, both proteins being found in the plasma bound to high-density lipoproteins (HDL) [6–10].

PON2 is a calcium-dependent glycoprotein of about 44 kDa, expressed ubiquitously [11], and associated with plasma membrane fractions [11, 12]. Recently it has been demonstrated that PON2 is a type II transmembrane protein, with its N-terminal region identified as a single transmembrane domain, whereas the catalytic domain, corresponds to the C-terminus, located extracellularly to counteract lipid peroxidation [13]. PON2 has been detected also in the perinuclear region, the endoplasmic reticulum ER and in mitochondria [14]; evidence has been provided suggesting that PON2 moves to the plasma membrane response to the Ca^++^-dependent oxidative stress [13]. PON2 has two main activities: a calcium-dependent hydrolytic activity, affecting mainly the hydrolysis of lactones, esters and aryl esters [15] and a redox function, which reduces the levels of ROS (reactive oxygen species) thus curbing cell oxidative stress and therefore displaying an anti-apoptotic effect [14]. In contrast with PON1 and PON3, PON2 does not show hydrolytic activity toward phosphotriesters, instead it mainly acts as a lactonase on homoserine lactones [15, 16]. Beside recent studies have shown that paraoxonases play an important role in counteracting biofilm formation during infection by pathogenic bacteria, which use quorum sensing molecules as a cell-density-dependent communication system [17, 18]. *Pseudomonas aeruginosa*, one of the most dangerous pathogens, uses 3OC12-HSL molecule as a quorum-sensing signal [19, 20]. Considering that murine epithelia cells not expressing PON2 show a reduced ability to counteract *P*. *aeruginosa* infection [17], it has been hypothesized that its physiological role may be attenuation of pathogens infection, such as *P*. *aeruginosa*, by *N*-(3-oxododecanoyl)-L-homoserine lactone (3OC12-HSL) hydrolysis.

Four putative *N*-linked glycosylation sites (Asn 226, Asn 254, Asn 269, Asn 323) have been predicted to be present in PON2, but only two have been experimentally validated: Asn 254 and Asn 323 [21, 22]. Glycosylation of these two sites is heterogeneous; in fact when the protein was analyzed by western blot it showed two distinct bands. Moreover it has been reported that the well-know polymorphism at position 311 (Ser/Cys) affects the level of glycosylation and drastically reduces the lactonase activity [21]. This result seems to be in contrast with Draganov and colleagues [15], reporting that recombinant PON1-3, produced using baculovirus/insect cell expression system, are glycosylated with high-mannose-type sugar, important for protein stability but not for hydrolytic activity. Furthermore it has been demonstrated that PON2 Asn254 and Asn 323 are glycosylated in EA.hy926 cells overexpressing the full-length PON2 protein and its mutants at positions 254 and 323, and these modifications have been demonstrated to be required for the enzyme hydrolytic activity [22]. However, differently from Stoltz and colleagues [21], no relation has been found between the polymorphism of residue 311 and the hydrolytic activity. Therefore, the different PON2 glycosylation pattern observed in different cell types and its correlation with the enzyme activity appears to be a matter worthy of further investigation. Horke and colleagues [23] found evidence to suggest the existence of a second post-translational modification of PON2 based on the relationship between hydrolytic activity and protein level in cells overexpressing PON2 upon treatment with 3OC12-HSL. In particular, it was observed that in A549 cells treated with 3OC12-HSL, PON2 expression level decreased by 50% in about 6 hours, whereas the residual hydrolytic activity was reduced to 25% after just 2 min compared with untreated cells. The same experiment with EA.hy929 cells showed a 50% reduction of hydrolytic activity after 10 minutes, indicating a different effect in different cell lines. In EA.hy926 cells a dose-dependent effect of 3OC12-HSL on PON2 hydrolytic activity was observed, and, interestingly, a decrease of PON2 protein level was detected up to 8 h after 3OC12-HSL treatment. The enzyme activity was rescued 5 hours after cell treatment, reaching about 80% of the initial value. These results seem to be consistent with a rapid and reversible modification [23].

In the present study we have analyzed the possibility to produce a recombinant PON2 in *E*.*coli* to better explore the main biochemical features of the enzyme, and above all we have focused on the relationship between PON2 hydrolytic activity and its post-translational modifications; in particular we have investigated the relationship between glycosylation and catalytic activity [21, 22], as well as the second modification postulated to modulate PON2 activity by Horke and colleagues [23].

## Materials and Methods

### Chemicals

All chemicals were reagent grade from Sigma Chemical Co. (St. Louis, MO, USA). Restriction enzymes were from New England BioLabs (Beverly, MA, USA).

### Molecular modelling

The PON2 3D model was generated from a structural alignment with PON1 and obtained from Swiss-Model, an automated server available at ExPASy web server (http://swissmodel.expasy.org). The X-ray crystal structure of the human PON1 (PBD code 1e04) was used as template. Only the sequence Leu16-Leu354 has been modelled. The model was reliable on the basis of the Ramachandran plot showing most of the residues in the core and allowed regions with the unique difference compared to PON1 represented by Pro 79 and Phe 106. The model was also tested with the program WHAT IF (http://swift.cmbi.ru.nl/server/html/index.html). The RMS deviation of the two superimposed structures was 0.25A on 331 alpha carbon atoms.

### Cloning and mutagenesis of *hPON2* open reading frame (ORF)

**Fig 1:**
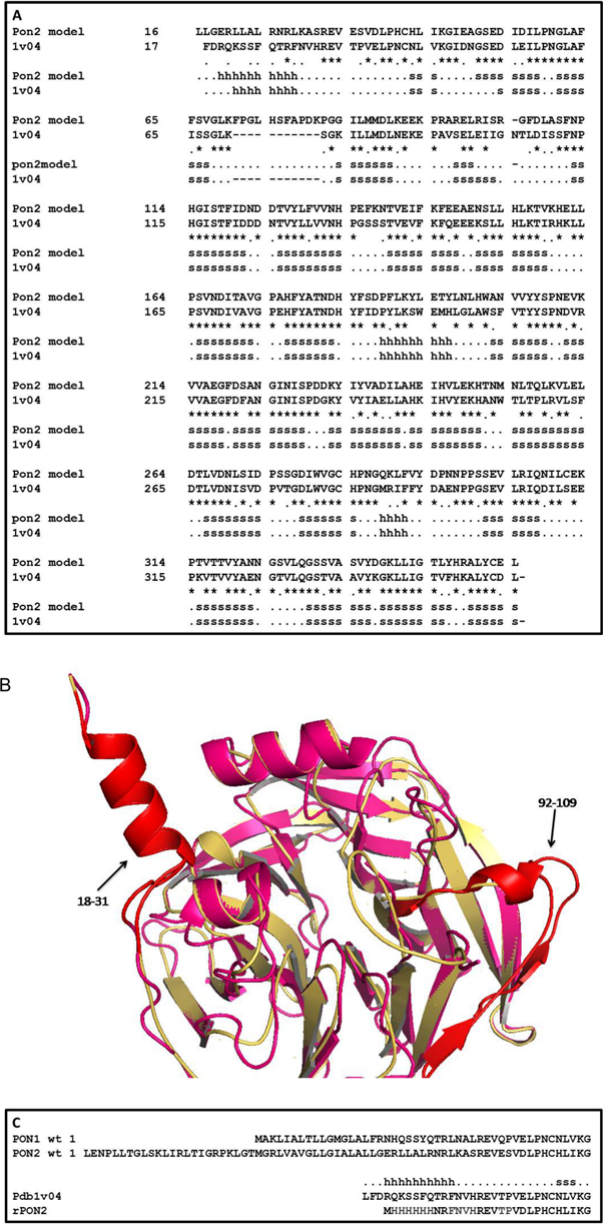
Structural sequences alignment between PON1 and PON2 (A) and their N-terminal regions (B). **(**A) Structural sequences alignment between PON1 (pdb:1v04) and PON2 3D model, with “h” and “s” indicating the alpha helix and the beta sheet regions respectively; the conserved residues are marked with asterisk. (B) Superposition between PON1 structure (cyan) and PON2 3D model (yellow), with highlighted by arrows regions 18–31 and 92–109 (red) mentioned in the text. (C) Sequences alignment of N-terminal regions of wild type PON1 and PON2 with the mutant version of crystallized PON1 (pdb:1v04) and the mutant version of PON2 that here was produced (rPON2); with “h” and “s” indicating the alpha helix and the beta sheet regions respectively. The His-tag added at the N-terminal of PON2 in place of the 49 N-terminal residues of the wild type, and the six residues mutagenized starting from the PON1 structure are indicated in grey.

The human PON2 full-length ORF cloned into pCDNA3, was kindly donated by Prof. S. Horke from Johannes Gutenberg University (Mainz, Germany) [22]. Starting from this clone, we mutagenized *hPON2* in three steps and sub-cloned it into the prokaryotic expression vector pT.7-7. In the first step, a 1017-bp fragment containing the *hPON2* ORF where residues 1–49 were substituted with a 6xHis-tag, was amplified (Fig 1A) using the pCDNA3/*hPON2* as the template, recombinant Taq DNA polymerase, and oligonucleotides *5’-PON2His* (5’-ggaaccttcatatgcatcatcatcatcatcataatcgatttaacgttcacaga-3’) and 3*’-PON2stop* (5’-acgcggatccttagagttcacaatacaaggc-3’) as forward and reverse primers, respectively, in a 30-cycle PCR (1 min 98°C, 1 min 45°C, 1 min and 20 sec 72°C). The amplification primer *5’-PON2His* was designed to introduce a *Nde*I restriction site (underlined) upstream of the initiation site, whereas *3’-PON2stop* was designed to introduce an *BamH*I restriction site (underlined) downstream of the stop codon of *hPON2*. The PCR product, digested with *Nde*I and *BamH*I, was ligated into the *Nde*I-*BamH*I-linearized expression vector pT7-7 (a derivative of pBR322, Stratagene) [24, 25] to create the pT7-7-*hPON2* construct. The ligation mixture was used to transform *E*.*coli* Top 10 (Invitrogen, CA, USA). The cloned fragment was completely sequenced to verify that only the desired mutations were introduced during the amplification procedure. The second step was to generate the double mutant E59T/S60P (Fig 1C), while starting from the double mutant the following quadruple mutations were added to obtain the variants L52F/K53N/A54V/S55H (Fig 1C). Both mutagenesis steps were carried as described by Pezzullo and colleagues [26], using the following complementary pairs of mutagenic oligonucleotides: *PON2-E59T/S60P* forward 5’-agagaagtaactccggtagaccttccacactgc-3’; *PON2-E59T/S60P* reverse 5’-tggaaggtctaccggagttacttctctggaggct-3’; *PON2-L52F/K53N/A54V/S55H* forward 5’-aatcgatttaacgttcacagagaagtaactccggt-3’; *PON2-L52F/K53N/A54V/S55H* reverse 5’-gttacttctctgtgaacgttaaatcgatttctgagtgcca-3’.

### Mutational analysis of Lys 168

Starting from the pT7-7-*hPON2* L52F/K53N/A54V/S55H/ E59T/S60P construct, and based on the mass spectrometric analysis, we mutagenized the residue Lys 168 (Fig 1A) in Ala and Arg. For these site-direct mutagenesis we use the QuikChange Lightning Site-Directed Mutagenesis Kit (Agilent Technologies, CA, USA), following the manufacturer's instructions, and as mutagenic primers the following complementary pairs of oligonucleotides were synthesized:

*PON2-K168A* forward 5’-gaatacagtggaaatttttgcatttgaagaagcagaaaat-3’;

*PON2-K168A* reverse 5’-attttctgcttcttcaaatgcaaaaatttccactgtattc-3’;

*PON2-K168R* forward 5’-gaatacagtggaaatttttcgatttgaagaagcagaaaat-3’;

*PON2-K168R* reverse 5’-attttctgcttcttcaaatcgaaaaatttccactgtattc-3’.

### Protein expression, *in vitro* refolding and purification

Five liters of LB medium containing 100 μg/ml of ampicillin were inoculated at an optical density (OD) of 0.005 (at 600 nm) and grown overnight at 37°C with vigorous bubbling of sterile air. The next day, after 3 hrs of IPTG induction (1 mM), cells were harvested by centrifugation (3,000 x *g*, 4°C, 10 min).

**Fig 2:**
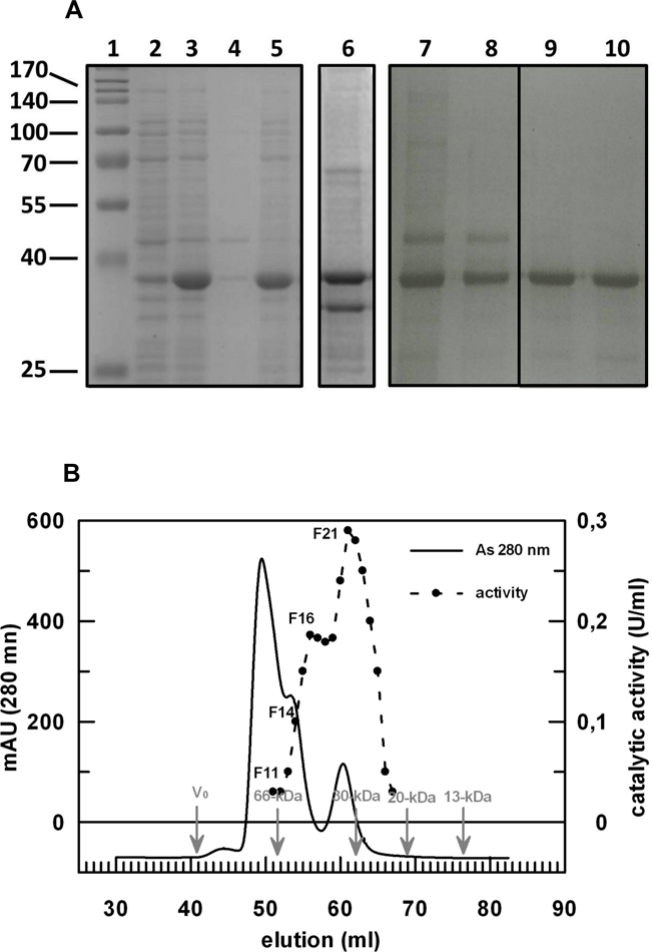
PON2 purification analysis by SDS-PAGE (A) and size-exclusion step on Superdex G-75 column (B). **(**A) SDS-PAGE analysis of the recombinant PON2 purification steps. Line 1: molecular weight markers (ProSieve QuadColor Protein Marker; Lonza, Rockland ME, USA); line 2: 0.05 O.D._600nm_ of a whole BL21(DE3)/pT7.7-PON2 cells; line 3: 0.05 O.D._600nm_ of a whole IPTG-induced (1 mM, 3h) BL21(DE3)/pT7.7-PON2 cells; line 4: soluble fraction derived from a BL21(DE3)/pT7.7-PON2 cells (0.05 O.D._600nm_) induced with IPTG (1 m, 3h); line 5: not soluble fraction derived from a BL21(DE3)/pT7.7-PON2 cells (0.05 O.D._600nm_) induced with IPTG (1 m, 3h); line 6: 5 μg of total proteins present in a fraction eluted from Ni-NTA; line 7: 20 μl of the fraction 11 (F11) which represent peak 1 (continuous trace, Fig 2B); line 8: 20 μl of the fraction 14 (F14) which represent peak 2 (continuous trace, Fig 2B); line 9: 20 μl of the fraction 16 (F16) showing the secondary peak of activity (dot trace, Fig 2B); line 10: 20 μl of the fraction 21 (F21) showing the maximum of activity (dot trace, Fig 2B) eluted by the size-exclusion column corresponding to peak 3 (continuous trace, Fig 2B). (B) last purification step of recombinant PON2 on 16/60 Superdex G-75 column (120 ml). Protein was followed by absorbance reading at 280 nm (continuous trace) or by activity measurements with the substrate *p*NP-propionate (dot trace). Molecular weight markers used for the gel-filtration 16/60 Superdex G-75 column calibration were: blue dextran (V_0_); ovalbumin (66 kDa); soybean trypsin inhibitor (30 kDa); lysozyme (20 kDa); Citochrome C (13 kDa) (GE Healthcare, USA).

All following procedures were carried out at room temperature, unless otherwise indicated. For the enzyme refolding and purification, wet frozen cells (1 g) were thawed and re-dissolved in 10 ml of buffer containing 20 mM Hepes pH 8.5; 0.5 mM CaCl_2_; 100 mM NaCl. Cell disruption was obtained by French pressure apparatus (Aminco Co., Silver Spring, MD, U.S.A.), using a pressure value of 2000 lb/in^2^ (1.38 MPa). Cell debris was removed by centrifuging (30,000 x *g*, 4°C, 20 min). The pellet was re-suspended in 10 ml of buffer containing 20 mM Hepes pH 8.5; 0.5 mM CaCl_2_; 5 mM glycine; 6 M urea; 50 mM β-mercaptoethanol, and incubated at room temperature for 6 hours to solubilize the inclusion bodies. The samples were diluted 100-fold in buffer A containing 20 mM Hepes pH 8.5; 0.5 mM CaCl_2_; 1 M urea; 0.1% (w/v) D(+)-trehalose and incubated for 24 hours at 4°C under gentle continuous stirring. Then 4 ml of Ni-NTA resin from Qiagen (Hilden, Germany) was added to the samples and incubated for 24 hours at 4°C and gently stirred continuously. The resin was loaded onto a column (20 ml), then washed with 50 ml buffer A without urea, plus 25 mM imidazole (buffer B) and eluted with buffer B (20 ml) containing 150 mM imidazole; the sample was recovered in fractions of 1 ml and the fractions were assayed. The fractions showing activity were pooled (about 15 ml total volume), concentrated by ultrafiltration onto a 10,000 cut-off cellulose membrane (from Amicon, Billerica, MA, USA) to 4 ml, divided in 2 ml aliquots) and loaded onto a Hi-Load 16/60 Superdex 75 column (GE Healthcare, USA; column volume 120 ml, recommended volume to be loaded is 2 ml). The column was equilibrated and eluted with buffer B containing 0.1 M NaCl. The flow rate was 0.5 ml/min. The fractions showing the highest specific activity, corresponding to the peak 3 (Fig 2B) were pooled and concentrated by ultrafiltration up to 2 ml; 2 mgs of pure enzyme were purified from 1 g of frozen cells. The same procedure was used to obtain the pure mutants K168A and K168R.

### Electrophoretic analysis

A 12.5% SDS-PAGE analysis was performed as described by Laemmli [27], at room temperature. “4.6 kDa-300 kDa ProSieve QuadColor Protein Marker” (Lonza, Rockland ME, USA) set was used as molecular weight standards.

### Steady-State Kinetic Measurements and Analysis

The time course of the catalysed hydrolysis of paraoxon, methyl-paraoxon (m-paraoxon), parathion, methyl-parathion (m-parathion), coumaphos, malathion, dursban, diazinon, *p*NP-esters and bis-*p*NP-phosphate (B*p*NP-P), was monitored essentially as described [28, 29]. Standard assays were performed with a Cary 100 double beam spectrophotometer (VARIAN, Australia) that automatically subtracted blanks, at 40°C, in a mixture of 20 mM Hepes buffer pH 8.5/0.5 mM CaCl_2_/4% acetonitrile, containing *p*NP-propionate (100 μM). The absorption coefficients at 405 nm used for *p*-nitrophenoxide were 20000 M^-1^ cm^-1^ at 40°C (pH 8.5). Initial velocities versus substrate concentration data were analyzed with the GRAFIT program (Grafit Version 3.0, Erithacus Software Ltd., UK). To determine of the specific esterase activities, *p*NP-proprionate was ranged from 0.025 to 2.0 mM. Kinetic parameters on TBBL (5-thiobutyl-γ-butyrolactone) were measured as reported [30]. Assays were carried out in duplicate or triplicate, and the results for the kinetic data were the average of two independent experiments.

### Lactonase activity

Enzyme activity toward Homoserine Lactones was measured by pH-tritation, using a pH-stat apparatus (T50 titrator model, Mettler Toledo, USA). The assays were done at 25°C, in 5 ml water adjusted to pH 8.5 with NaOH 0.1 M, containing 2% acetonitrile and 0.5 mM CaCl_2_. Stock solutions of C4-HSL and 3OC12-HSL were prepared by dissolving pure lactones in acetonitrile. Kinetic parameters were measured with substrate concentrations ranging from 0.1 to 1.2 mM; for each point, the blank was measured and subtracted. Assays were done in duplicate or triplicate and results are the average of two independent experiments. The activity (V_*max*_) was defined as μeq min^-1^mg^-1^, which is the quantity of NaOH used to neutralize the free acids developed from enzymatic reaction in one minute by 1 mg of rPON2.

### Inhibition of biofilm formation of *Pseudomonas aeruginosa* by rPON2

In this experiment *Pseudomonas aeruginosa* PAO1 was used as a reference strain. Bacteria were grown in Mueller-Hinton medium (solid and liquid) at 37°C. Cells stored at– 80°C were spread onto Mueller-Hinton solid medium and grown 16 h at 37°C; colonies were transferred in liquid medium and incubated overnight at 37°C under constant shaking. The next day cells were diluted in fresh Mueller-Hinton liquid medium and grown at 37°C with shaking up to 0.2 O.D._600nm_, then the cells were inoculated in a 96-well microplate in the presence or absence of rPON2 (50 and 100 μg/ml), PON1 (100 μg/ml), and incubated at 37°C in a humid atmosphere for 48 h. PAO1 was used as control in the presence or not of the enzyme buffer (20 mM Hepes pH 8.5 containing 0.5 mM Ca^++^). After incubation, the medium was removed from the wells, and wells were washed three times with distilled water. The crystal violet staining was performed essentially as described by Nagant and colleagues [31]; the absorbance of each well was read at 540 nm in a Victor3 PerkinElmer 1420 Multilabel counter (PerkinElmer, Waltham, MA, USA). Each condition was replicated in six different wells and the experiment was carried out twice.

### Western blot analysis

After purification of rPON2 incubated in HeLa extract, the enzyme was run on SDS-PAGE gel electrophoresis (500 ng of purified enzyme for each line), the protein was electro-transferred (1 h at 100 V) onto a polyvinylidene difluoride immunoblot membrane (PVDF, 0.2 μ) using a transblot apparatus (BioRad, USA) in 0.375 M Tris-Glycine buffer (pH 8.3) containing 10% methanol. The membranes were blocked by incubation with milk 5% (w/v) (nonfat dried milk powder, Applichem, Germany, catalog number A0830,0500) in 1x phosphate-buffered saline containing Tween 20 (0.05% v/v) and incubated for 1 h with an anti-PON2 mouse monoclonal antibody (catalog number ab85340 AbCam, Cambridge, UK), or an anti-phosphothreonine mouse monoclonal antibody (catalog number 032M4789, Sigma-Aldrich, USA), or an anti-phosphotyrosine mouse monoclonal antibody (catalog number 051M4831, Sigma-Aldrich, USA), or an anti-phosphoserine mouse monoclonal antibody (catalog number 111M4763, Sigma-Aldrich, USA), respectively. After washing, the membranes were incubated for 1 h with horseradish peroxidase-conjugated goat anti-mouse IgG (catalog number 115035003, Jackson ImmunoResearch, West Grove, PA, USA). The membranes were developed with a chemiluminescent substrate (ECL kit from AbCam, Cambridge, UK, catalog number ab65628).

### rPON2 activity in HeLa extract

Freshly prepared rPON2 and engineered mutants were added to HeLa extracts, to test their enzymatic activity and the protein level after incubation. The cells were grown in DMEM medium (Life Technologies, Carlsbad, CA, USA) supplemented with 10% FBS (Euroclone, Milan, Italy), 100 U/ml penicillin and 100 μg/ml streptomycin (Sigma-Aldrich, USA). Three cell extract samples were prepared by lysing cells in RIPA Buffer containing protease inhibitors (Sigma-Aldrich, USA). The total proteins present in the extract were quantified (2.5 mg/ml), and the freshly purified rPON2 or K168 mutants were added to a final concentration of 0.1 mg/ml. Aliquots of 25 μg of total proteins (containing about 1 μg of rPON2) were withdrawn at 0, 15, 30, 45 and 60 min of incubation at 37°C. These aliquots were analyzed for their catalytic activity (measured on *p*NP-C3 in standard assay) and by western blot using antibodies against PON2. HeLa cell extract was obtained starting from culture samples of about 70% of cell confluence. The same experiment was repeated using HeLa cells treated with 3OC12-HSL added to the medium for 10 minutes, as previously reported [23].

### Mass spectrometric analysis

To identify putative post-translational modification on PON2 that affected the catalytic activity, we incubated freshly purified rPON2 and the mutants K168A and K168R in HeLa extracts under the conditions cited above. After 30 minutes of incubation, the enzymes were re-purified by Ni-NTA affinity column and separated onto 12.5% SDS-PAGE; as a control pure rPON2 not incubated in HeLa cell extracts was loaded. The rPON2 bands were cut and analyzed by LC-MS/MS (Central Proteomics Facility-Sir William Dunn Pathology School- South Parks Rd, Oxford, OX1 3RE). From the analysis, we obtained 90% coverage of the PON2 sequence from residues 68 to the 369 (Fig 1A).

## Results and Discussion

### PON2 modelling and expression

A first attempt to express in *E*.*coli* the full length PON2 gene from the pCDNA-3 HIS vector, by exploiting a His-tag appended at the C-terminus failed, the protein appearing partially degraded. A Western blot performed using an antibody against the His-tag gave a positive reaction suggesting that a peptide fragment was likely missing from the N-terminus (data not shown). In order to design a more putatively stable PON2 version, a 3D model was built on the basis of the 3D structure of the homolog PON1 (PDB code: 1v04) [32]. The modelling was performed by using the tools available under the Swiss Pdb Server, as reported in Methods (Fig 1A and 1B).

The 3D structure of PON1 (and PON2) is characterized by the first α-helix at the N-terminus that is apparently exposed; the N- and C-terminal domains are covalently linked by a disulfide bridge between Cys42 (strand 6D) and Cys353 (strand 6C) (Fig 1A) [32] likely conserved in PON2. The loop between the strand D6 and the N-terminal α-helix is involved in the structural stabilization of PON1 by several interactions [32]. Looking at the PON2 model (Fig 1B), the regions 18–31 and 92–109 were quite dissimilar to PON1. For these reasons we substituted six residues in PON2 with residues that were found to stabilize PON1.

Based on the model analysis and data from literature concerning PON1 [32], an engineered version of the PON2 ORF was inserted into the expression vector pT7-7, with the His-tag placed at the N-terminus for these studies. Using this approach we intended to remove the highly hydrophobic N-terminal region of PON2, because in preliminary experiments of protein expression, the full length protein was not soluble, probably due to the hydrophobic N-terminal region that is involved in membrane interaction. We substituted this region with a 6-HisTag, to have the tag outside of the protein structural core. Finally, six residues (E59, S60, L52, K53, A54, S55) were changed with those found stabilizing the mutated form of PON1 [32] (Fig 1A). The six substitutions introduced were: L52F/K53N/A54V/S55H/E59T/S60P (Fig 1C). The new N-terminal sequence of the recombinant PON2 is reported in Fig 1B, along with the structural alignment of wild type PON1 and PON2 model. The predicted molecular weight of the rPON2 was 37843.8 Da.

The correct expression of rPON2 was confirmed by detection of a polypeptide of the predicted size and by a western blot analysis using antibodies directed against both the 6-His tag and an internal region of human PON2. Unfortunately, the recombinant protein was found exclusively in the inclusion bodies.

At this point we set-up a procedure (see Methods) to extract and to refold *in vitro* the enzyme starting from the inclusion bodies. The optimized procedure resulted in the generation of a fully active rPON2. The specific activity measured on *p*NP-propionate was comparable to that measured for the full-length protein expressed in insect cells by Draganov and colleagues [15]. Briefly, of frozen cells (1 gr) overexpressing rPON2 were disrupted by a French pressure apparatus; the insoluble fraction, containing the inclusion bodies, was recovered by centrifugation and solubilized by urea and β-mercaptoethanol incubation. The *in vitro* refolding was made by 100-fold dilution in a buffer containing CaCl_2_ (0.5 mM) (because the PON2 activity is calcium dependent), and D(+)-trehalose (0.1%, w/v) to increase the enzyme stabilization during the in vitro folding [33]. The His-tagged rPON2 was recovered by a Ni-NTA affinity resin (Qiagen, Germany) and eluted by imidazole (150 mM). The fractions showing activity were pooled and concentrated through ultrafiltration. All the purification steps were followed by SDS-PAGE analysis (Fig 2A). The enzyme was further purified to homogeneity by preparative size-exclusion chromatography on a 16/60 Superdex G-75 (GE Healthcare, USA) column (Fig 2B). SDS-PAGE analysis of the peaks obtained by the size-exclusion chromatography revealed that peak 1, eluting at a volume corresponding to a molecular mass of about 70 kDa, contained contaminant proteins and rPON2 as a dimer (Fig 2A, line 7); the specific activity of the rPON2 present in this fraction (F 11) was 0.008 U/mg, measured on *p*NP-propionate. Peak 2, eluting at a volume corresponding to a molecular mass of about 60 kDa, contained contaminant proteins and rPON2 as an asymmetric dimer (Fig 2A, line 8); the specific activity of the rPON2 present in this fraction (F 14) was 0.08 U/mg. Peak 3, eluted at a volume corresponding to a molecular mass of about 35 kDa containing pure monomeric rPON2. The specific activity measured was 0.015 U/mg (F 21). An intermediate value of specific activity was measured for the fraction F 16 (0.10 U/mg) which is intermediate between the peak 2 and 3, indicating an improperly folded rPON2 in this fraction. The fully active rPON2 corresponds to a monomeric form (peak 3), in agreement with PON1 that appears to be monomeric based on the refined crystal structure containing one molecule per asymmetric unit [32]. The amount of rPON2 produced in *E*.*coli* was estimated to be about 50 mg/g of frozen cells, whereas the procedure that was set up to fold the enzyme *in vitro* allowed us to obtain about 20 mg of soluble rPON2. However in the last step of purification (size-exclusion chromatography) we separated the monomeric fully active form of rPON2 from the dimeric and incorrectly folded enzyme -showing lower specific activity- with a final yield of 2 mg of pure fully active enzyme.

### Substrate specificity

In order to compare rPON2 with the full-length enzyme, characterized by Draganov and colleagues [15], we analyzed (Table 1) the catalytic activity using various classes of substrates, such as *p*NP-esters with different acyl chain length (from 2 to 10 carbon atoms), pesticides organophosphates, a phosphodiester, a galactoside and the latone TBBL. We observed that the best substrate was TBBL; this is in contrast with a recent paper published by Bar-Rogovsky and colleagues [34] reporting that TBBL is not a substrate for PON2. However the enzyme used by Bar-Rogovsky et al., was the wild type full-length version. In contrast, our version lacks the N-terminal region, which is not important for the catalytic activity [13], and presents six point mutations at the new N-terminal region. Moreover we detected two new secondary activities on the phosphodiester Bis-*p*NP-phosphate and the galactoside *p*NP-α-L-arabinopyranoside. In contrast with the data previously reported in literature, Bar-Rogovsky and colleagues [34] detected activity on m-paraoxon using a full-length version of PON2 expressed with the baculovirus/insect cell expression system. Strangely enough, we did not detect this activity consistent with previously reported results [15], but we observed activity on organophosphorothiote pesticides, m-parathion (0.33 U/mg), coumaphos (0.014 U/mg) and malathion (0.016 U/mg) (Table 1). Although we cannot exclude that these differences could be due to the mutations introduced in our PON2 version, it should be considered that we used a high amount of protein and substrates and it is likely that under these conditions we detected promiscuous activities that were neglected by others. Because Bar-Rogovsky and colleagues propose to use such differences in activity as a tool to discriminate between PONs, this issue needs to be more thoroughly analyzed. Among carboxylesters, the best substrate for our PON2 version was *p*NP-propionate (Table 1), confirming that rPON2 represents a good model of the native PON2.

**Table 1:** Substrate specificity of the purified recombinant human PON2.

Class	Substrate	Specific activity (U/mg)
**Lactones**	**TBBL**	**1.40 ± 0.07**
**Esters**	***p*NP-acetate (C2)**	**0.28 ± 0.03**
	***p*NP-propionate (C3)**	**0.57 ± 0.05**
	***p*NP-butirate (C4)**	**0.20 ± 0.04**
	***p*NP-valerate (C5)**	**0.24 ± 0.02**
	***p*NP-caproate (C6)**	**0.19 ± 0.02**
	***p*NP-octanoate (C8)**	**0.16 ± 0.01**
	***p*NP-decanoate (C10)**	**0.11 ± 0.01**
**Phosphodiesters**	**Bis-*p*NP-phosphate**	**0.036 ± 0.004**
**Glucosides**	***p*NP-α-L-arabinopyranoside**	**0.039 ± 0.004**
**Pesticides**	**paraoxon**	**n.d.**
**organophosphate**	**m-paraoxon**	**n.d.**
**Pesticides Organophosphothiotes**	**parathion**	**n.d.**
	**m-parathion**	**0.33 ± 0.03**
	**coumaphos**	**0.014 ± 0.003**
	**malathion**	**0.016 ± 0.002**
	**dursban**	**n.d.**
	**diazinon**	**n.d.**

Assays were done in 20 mM Hepes pH 8.5 + Ca^++^ 0.5 mM, 40°C, and at specific concentration of substrate as reported in Materials. Each point is the mean of two independent experiments with two different enzyme preparations.

**n.d.** = not detected, no activity beyond background rate was detected.

After this initial set of experiments, we decided to measure the kinetic parameters only for the substrates that were hydrolysed more efficiently. The rates of hydrolysis of the synthetic lactone TBBL and *p*NP-propionate were measured spectrophotometrically (Table 2). The two lactones C4-HSL and 3OC12-HSL, relevant from a physiological point of view [23], were tested by pH-titration, *p*NP-propionate was measured as control (Table 3). The comparison of activities by pH-titration confirmed that the best substrate was 3OC12-HSL, as reported for the full- length PON2 expressed in insect cells [34].

**Table 2:** Kinetic parameters of the purified rPON2 and K168 mutants.

enzymes	substrate	*k*_cat_ (sec^-1^)	K_M_ (mM)	*k*_cat/_ K_M_ (sec^-1^ mM^-1^)
**rPON2**	***p*NP-propionate**	**1.27±0.13**	**0.90±0.20**	**1.41±0.18**
	**TBBL**	**1.10±0.10**	**0.50±0.15**	**2.20±0.50**
**K168A**	***p*NP-propionate**	**0.40±0.04**	**1.50±0.25**	**0.27±0.07**
**K168R**	***p*NP-propionate**	**1.67±0.26**	**1.20±0.30**	**1.40±0.10**

The data in the table were determined from spectrometric assays at 40°C, in 20 mM Hepes pH 8.5 containing 0.5 mM Ca^++^. Results are mean of two independent measurements.

**Table 3:** Kinetic parameters of the purified rPON2 and K168 mutants.

enzyme	substrate	V*max* (μeq min^-1^ mg^-1^)	K_M_ (mM)
**rPON2**	**3OC12-HSL**	**4.1±0.4**	**0.5±0.1**
	**C4-HSL**	**2.3±0.2**	**0.6±0.1**
	***p*NP-propionate**	**1.4±0.2**	**0.9±0.2**
**K168A**	**3OC12-HSL**	**1.5±0.2**	**0.9±0.2**
**K168R**	**3OC12-HSL**	**5.5±0.3**	**0.6±0.2**

The values reported in the table were determined by pH titration at 25°C, using as substrates 3OC12-HSL for all the enzymes and C4-HSL and *p*NP-propionate only for rPON2. Results are the average of two independent measurements.

In conclusion and in contrast with results previously reported which indicated that the PON2 activity is strictly dependent on its glycosylation [21, 22], we obtained a fully active enzyme despite producing a non-glycosylated rPON2 in *E*.*coli*. Based on the results of increased stability of rPON2 after addition of threalose to the storage buffer (S4 Fig), we concluded that glycosylation does not affect the catalytic activity of PON2 but its stability. In the case of PON1 the enzyme has been expressed as a GST-fused protein in *E*.*coli* expression system [38]. The resulting enzyme was soluble, catalytically active and stable despite to the fact that it was not glycosylated [38].

Similar results were reported in a previous paper [39] in which by chemical modification and site-directed mutagenesis glycosylation of residues Asn 252 and 323 was found to be not essential for PON1 secretion and activity [39]. Our results too indicate that PON2 stability is related to enzyme glycosylation, in agreement with the general notion that glycosylation confers stability against protein aggregation while enhancing thermal stability [40–42].

### Inhibition of biofilm formation

**Fig 3:**
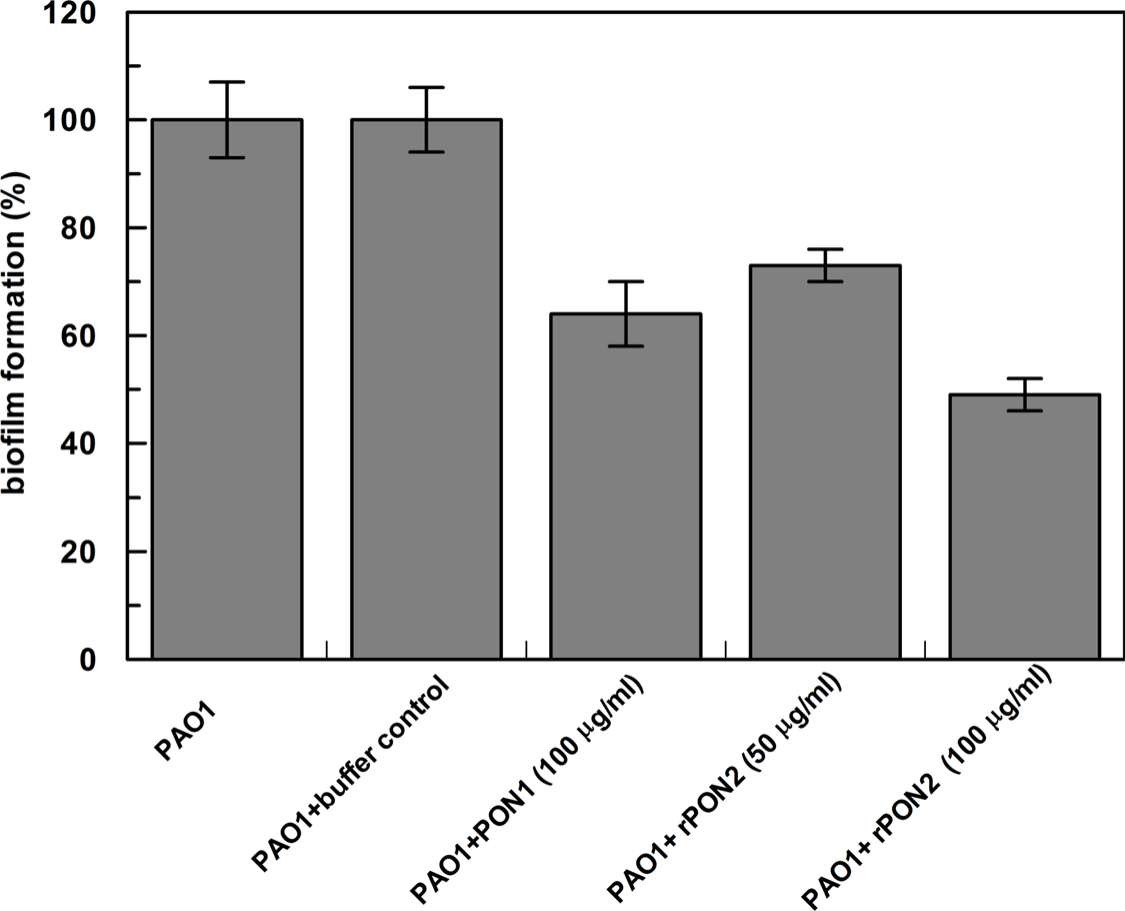
Biofilm formation. The PAO1 cells were grown o.n. and inoculated in a 96-well microplate in the presence and absence of rPON2 (50 and 100 μg/ml) and PON1 (100 μg/ml). PAO1 cells grown in presence of the enzyme storage buffer (20 mM pH 8.5 containing 0.2 mM Ca^++^) were used as control. After 48 h of incubation at 37°C, the medium was removed from the wells, and the biofilm formation was evaluated by crystal violet assay. The absorbance of each well was read at 540 nm. Biofilm formation was reported as a percentage in comparison with to the maximum amount of biofilm produced by POA1 cells grown in absence of enzymes (positive control). Each condition was made in six different wells and the experiment was carried out twice. Results are the average of duplicate and error bars show the range of the duplicate.

As previously reported [17, 35], PON2 displays the highest activity on homoserine lactones, in particular on 3OC12-HSL, a signal molecule exploited by gram-negative pathogens, like *P*. *aeruginosa* (PAO1), in the mechanism of Quorum Sensing [36], a microbial system which regulates the virulence and biofilm formation. For this reason, we tested the capability of our rPON2 to inhibit the formation of PAO1 biofilm *in vitro*. The inhibition was evaluated by the crystal violet method [37], after 48 h of incubation of PAO1 in 96-well plates in the presence of the enzymes and/or the same amount of buffer in which the enzymes were solubilized, as controls. PAO1 grown in the presence or absence of buffer formed the same amount of biofilm, whereas in the presence of lactonases a decrease in biofilm formation was observed. In particular, PON1 at 100 μg/ml generated about 35% inhibition, whereas rPON2, added at 50 and 100 μg/ml, yielded about 25 and 50% of inhibition respectively, showing a higher capability to modulate biofilm formation (Fig 3).

### rPON2 activity in HeLa cell extract and identification of a post-translational modification

In a recent paper [23], based on mRNA and hydrolytic activity analyses, it was postulated that PON2 under certain conditions is affected by a rapid (less than 20 minutes) post-translational modification, which dramatically decreases the hydrolytic activity without modifying the total amount of PON2 protein present in the cell extract. Until now, this putative modification remained elusive, but given the significant amounts of rPON2 which were available to us, we decided to approach its identification at molecular level. In detail, freshly purified rPON2 was firstly analysed for phosphorylation by western blot experiments using specific antibodies against phospho-residues before and after 20 minutes of incubation in a HeLa cell extract treated with or without 3OC12-homoserine lactone (see Materials and Methods for western blot conditions); in correspondence of rPON2 band, no phosphorylation signal was detected and no differences in the phosphorylation level was recorded after incubation in a HeLa cell extract, suggesting that phosphorylation was not involved in the mechanism of PON2 activity regulation.

With the aim of reproducing *in vitro* the experiment of Horke *et al*. [23], purified rPON2 was added to HeLa cell extracts treated with or without 3OC12-homoserine lactone and incubated up to 60 min at 37°C.

This experiment was aimed at: *a*) measuring the catalytic activity of rPON2 before and after incubation with HeLa cell extract (treated with 3OC12-homoserine lactone) for different incubation times; and *b*) recovering rPON2 from HeLa cell extract (treated with 3OC12-HSL) by Ni-affinity chromatography to re-analyze the catalytic activity and protein modifications by mass spectrometry.

**Fig 4:**
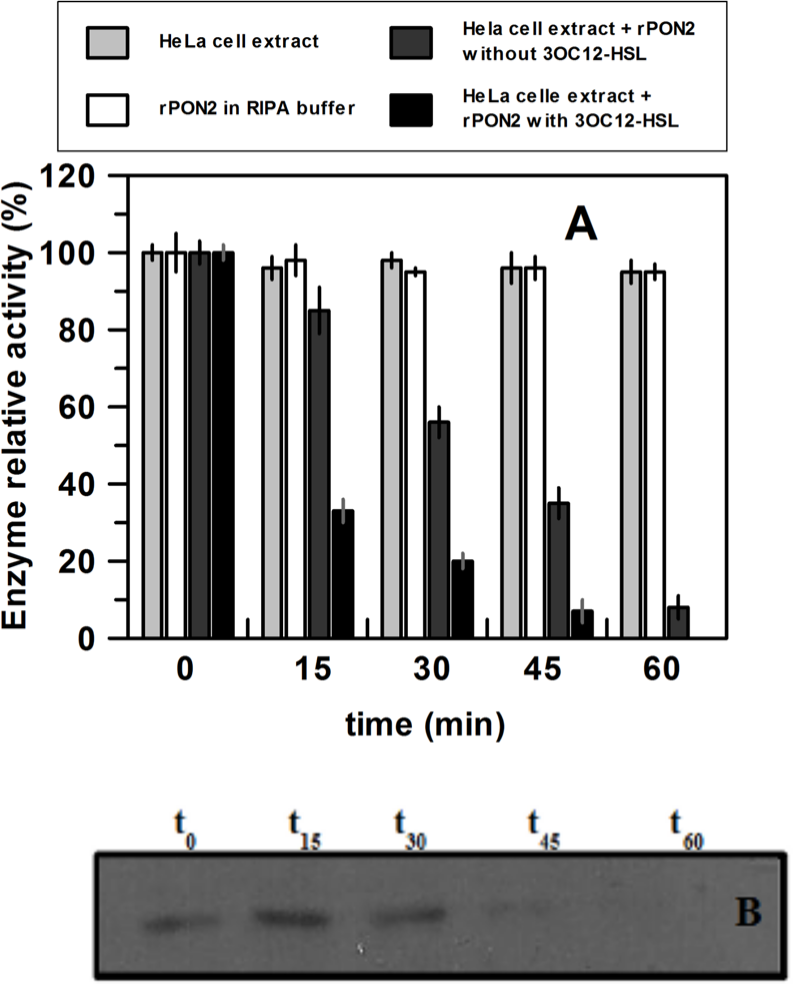
Esterase activity (A) and western blot analysis (B) of rPON2 in HeLa cell extracts. (A) Purified rPON2 was tested for its esterase activity in pure form, diluted in RIPA buffer, and mixed in HeLa cell extracts with or without 3OC12-HSL. The catalytic activity was followed in intervals ranging from 0 to 60 minutes. The assays were carried out with *p*NP-propionate as a substrate at 40°C. The activities were reported as residual activity (%) relative to the initial value measured. Results are the average of duplicate and error bars show the range of the duplicate. (B) Western blot analysis of rPON2 in HeLa cell extracts with 3OC12-HSL. Samples were withdrawn at the same time interval used to test the catalytic activity. By densitometric analyses (GelQuantNET, available at biochemlabsolution.com) the amount of rPON2 present in HeLa cell extracts was measured: at time t_0_ we considered the signal corresponding to the rPON2 as 100%, at 15 min the relative signal was 115%, at 30 min 108%, at 45 min 25% and after 60 min no signal was revealed.

Therefore rPON2 was added at 0.1 mg/ml in a HeLa cell extract, and the activity was measured at time = 0 (t_0_) roughly corresponding to the sum of the blank activity (HeLa cell extract treated with 3OC12-HSL) and the rPON2 activity. After 30 minutes of incubation, the level of activity was less than 20% (Fig 4A) but by western blot analysis we did not observe any significant decrease of protein (Fig 4B). Controls consisting of HeLa cell extract supplemented with the rPON2 buffer only and rPON2 diluted in RIPA buffer, gave no difference in activity with incubation up to 60 minutes. The same was true for rPON2 incubated with the HeLa cell extract without 3OC12-HSL (Fig 4A). Therefore we were able to completely reconstitute *in vitro* the result of a fast decrease of PON2 activity when the enzyme was expressed in eukaryotic cells treated with 3OC12-HSL, without reduction of PON2 protein level [23].

Then we re-purified rPON2 from HeLa cell extract treated with or without 3OC12-HSL by Ni-NTA column affinity (Qiagen, Hilden, Germany), and these samples were used for the mass spectrometry analysis to identify post-translational modified residues by comparison with a freshly purified enzyme. The samples were digested with trypsin, peptides fractionated on nanoHPLC and analyzed by ESI-MSMS on a Thermo Orbitrap mass spectrometer. The analysis showed that the rPON2 incubated in HeLa cell extract treated with 3OC12-HSL was post-translationally modified via ubiquitination of Lys 168. The peptide identified by mass spectroscopy containing the ubiquitinated Lys was NTVEIF**K**FEEAENSLLHLK (Lys 168 is indicated in bold).

**Fig 5:**
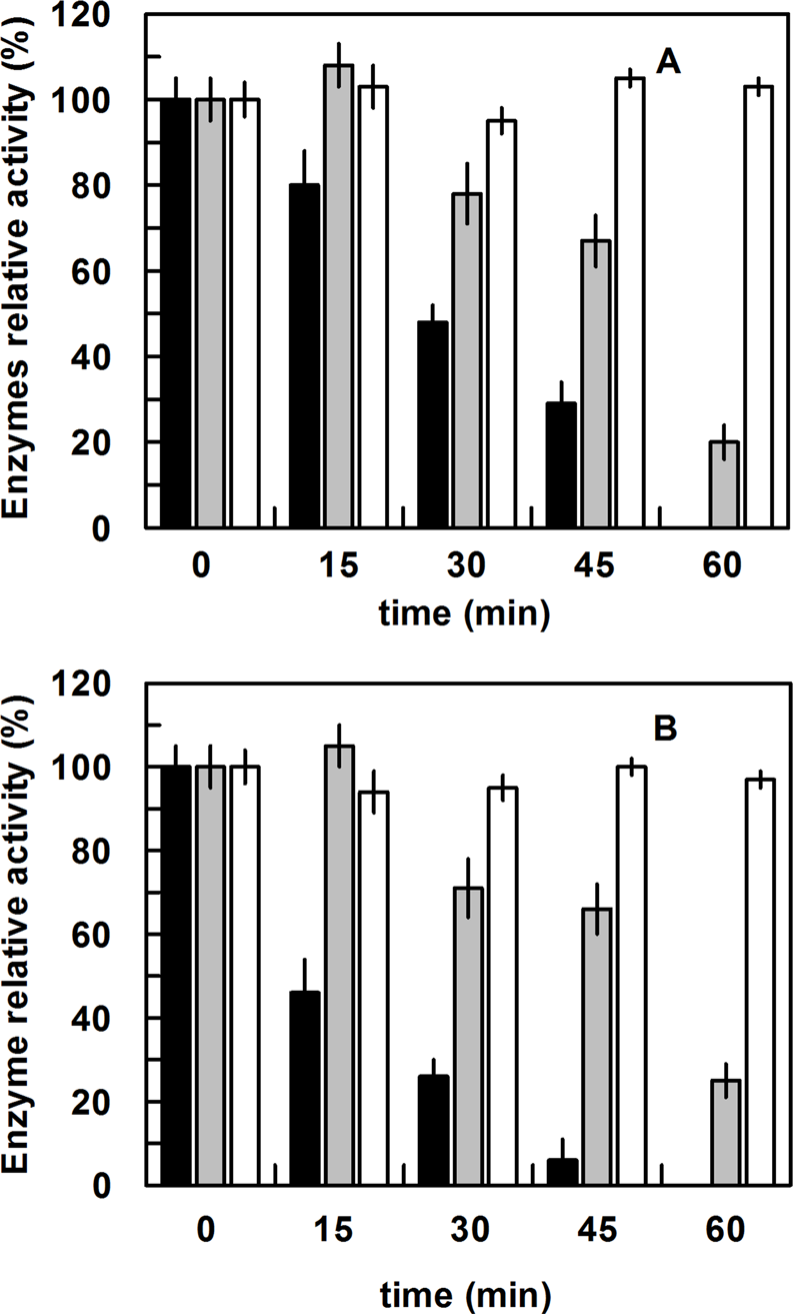
rPON2 and K168 mutants activity in HeLa cell extracts without (A) or with (B) 3OC12-HSL. Purified enzymes were tested for their esterase activity in pure form or mixed with HeLa cell extracts, following the activity in intervals of time ranging from 0 to 60 minutes. The assays were carried out at 40°C using *p*NP-propionate as substrate. The activities were reported as residual relative activity (%) relative to the initial value measured. The rPON2 was reported in black, the mutant K168A in grey, and the mutant K168Rin white. (A) The incubation was made in HeLa cell extract not treated with 3OC12-HSL; (B) The incubation was made in HeLa cell extract treated with 3OC12-HSL (100 μM for 10 minutes). As controls, HeLa cell extracts supplemented with the rPON2 buffer only and rPON2 diluted in the RIPA buffer were used and no differences in activity were recorded up to 60 min incubation (data not show). Results are the average of duplicate and error bars show the range of the duplicates.

Ubiquitination is a mechanism involved in fundamental cellular events such as protein degradation, cell-cycle regulation and DNA repair, and in literature many papers have reported ubiquitination as a mechanism of enzyme activity regulation [43, 44]. Therefore the next question was if this modification affected the catalytic activity of rPON2. In order to shed light on this point, we generated two mutants at the position 168: K168A and K168R. The two variants were expressed, refolded and purified as rPON2. Firstly, we measured the optimal pH and temperature of these new mutants, observing that these parameters were unchanged compared to rPON2; then we analyzed their thermal stability (S3 Fig) observing that the mutant K168R was more stable than rPON2 and K168A. From a kinetic point of view, both new mutants exhibited the best activity with the lactone 3OC12-HSL (Table 3), but the mutant K168R showed an increased activity with respect to rPON2 and the K168A mutant, when assayed with *p*NP-propionate as substrate (Table 2). These results allowed to rule out any negative effect of the mutations on the overall structure of the enzyme. We repeated with all the mutants the experiment incubating the purified enzymes in HeLa cell extracts with or without 3OC12-HSL. The results reported in Fig 5A showed for rPON2 a decrease in activity when it was incubated in HeLa cell extract without 3OC12-HSL (50% of residual activity after 30 min and no activity detected after 60 min), in agreement with previous experiment. The mutant K168A showed a decrease in activity (80% after 60 min), whereas the mutant K168R did not show any decrease in activity up to 60 minutes of incubation in a HeLa cell extract. The decrease was more dramatic than expected when HeLa cells were treated with 3OC12-HSL (Fig 5B), in fact for rPON2 after 15, 30 and 45 minutes incubation about 50-25- and 0% of residual activity were measured, respectively. The mutant K168A showed the same pattern of residual activity compared to that obtained by incubation in HeLa cell extract without 3OC12-HSL treatment, and again the mutant K168R did not show a decrease in activity after 60 min of incubation in HeLa cell extract treated with 3OC12-HSL. Since both mutants K168A and K168R are resistant to ubiquitination, the decrease in activity observed in the mutant K168A was due to mainly a reduced stability of this mutant compared to the wild type, as observed in the experiment of thermal stability measurement, in which at 40°C the pure mutant K168A showed a half-life (*t*_1/2_) of about 150 min with respect to the *t*_1/2_s of 240 min and more than 300 min for the rPON2 or the mutant K168R respectively (S3A and S3B Fig). This reduced stability can be interpreted in terms of a conformational change induced by the mutation (above all we changed a large and charged residue with a small and hydrophobic one!) that in turn affects the activity. Instead we could explain the increase of stability of the mutant K168R compared to rPON2 by a better stabilization of the Arginine charge with respect to the Lysine residues; in fact it is well known the high hydrogen-bonding ability of Arginine with respect to Lysine. These results indicated that rPON2 decrease in activity due to incubation in the HeLa cell extract was accelerated when treated with the 3OC12-HSL, while the activity of the mutant K168R was not affected by incubation with the lactone. Since this mutant was not susceptible to the ubiquitination at the position 168, we believe this to be a direct *in vitro* evidence that this post-translational modification was responsible for the decrease in rPON2 activity.

Our approach has a limitation however because we were unable to discriminate between mono- or poly-ubiquitination. This will be a matter of future work. In a previous paper [45], focusing on the ubiquitin modified cellular proteome, it was found that PON2 is ubiquitinated at Lys 313; this finding was just an annotation without any indication of the putative physiological or catalytical relevance of this modification. In our analysis, we did not observe the postulated ubiquitination at Lys 313.

## Conclusions

Here we report a method to produce an engineered version of the human PON2 in *E*.*coli*, with the aim of studying its catalytic properties, and the relationship between PON2 activity and post-translational modifications. Regarding the catalytic activity, we confirmed that the best substrate was 3OC12-HSL (Tables 1–3), in full agreement with the *in vitro* evidence of inhibition of PAO1 biofilm formation; the recombinant PON2 behaved better the other human paraoxonase PON1 (Fig 3), indicating that its physiological role could be the attenuation of infection of some pathogens by 3OC12-HSL hydrolysis, as previously hypothesized [17].

A second post-translational modification was postulated to influence the PON2 activity [23]. We analyzed this aspect by enzyme incubation in Hela cells extract with or without 3OC12-HSL, followed by mass spectrometric analysis, observing as the only modification the ubiquitination of the Lys 168.

We examined the relationship between this post-translational modification and the PON2 catalytic activity by mutational analysis- Both mutants produced, K168A and K168R, were not susceptible to this modification and their catalytic activity did not change when the enzymes were incubated in HeLa cell extract with or without 3OC12-HSL (Fig 5A and 5B), confirming the relationship between ubiqutination of Lys 168 and PON2 catalytic activity.

Our results indicated that PON2 Lys 168 ubiquitination is part of the mechanism that is responsible for the enzyme activity modulation by 3OC12-HSL. Many aspects require further investigation. In particular, of paramount importance will be the identification of the pathways activated by the 3OC12-HSL that promote the ubiquitination of PON2. The knowledge of this mechanism may be a basis for counteracting infections by pathogens like PAO1.

## Supporting Information

S1 Fig Determination of the optimal temperature for esterase activity. Activity was measured over the range 20–55°C, using the standard esterase assay, *p*NP-propionate was used as substrate. The dependence of enzymatic activity on temperature was studied over the range of 20–55°C, using *p*NP-propionate in the standard assay conditions. The optimal temperature was confirmed also for the lactonase activity on TBBL.
(PDF)

S2 Fig Determination of the optimal pH for esterase activity. Assays were done at 40°C, using *p*NP-propionate as substrate. Buffer used were: 20 mM Na_2_HPO_4_/NaH_2_PO_4_ over the range 6.5–7.5 (*circles*); 20 mM Tris/HCl over the range 7.5–8.5 (*squares*); 20 mM Hepes over the range 8.5–9.5 (*triangles*). The assays were carried out in duplicate or triplicate and the results were the means of two independent experiments. The optimal pH was confirmed also for the lactonase activity on TBBL.
(PDF)

S3 Fig Thermal stability. Determinations were made after incubation at 40°C (A) and 50°C (B). The thermal stability of rPON2 and its mutants was assayed at 40 and 50°C. Pure enzymes (0.2 mg/ml in 20 mM Hepes pH 8.5 containing CaCl_2_ 0.5 mM) were incubated in seals glass tubes. Aliquots were withdrawn at time and assayed at 40°C in standard esterase assay.
(PDF)

S4 Fig Enzyme stability. rPON2 at 0.2 mg/ml was dialysed against 20 mM Hepes pH 8.5 containing (open circles) urea 0.25 M and D(+)-trehalose (0.1%) or not (full circles), at 4°C for 12 days. The activity was reported as residual activity respect to the value measured at t_0_ of incubation. The stability of rPON2 was studied maintaining the enzyme (0.2 mg/ml) at 4°C in storage buffer 20 mM Hepes pH 8.5 containing 0.5 mM CaCl_2_ /0.25 M urea/ 0.1% (w/v) D(+)-trehalose and in dialysis against storage buffer with or without urea and D(+)-trehalose. Aliquots were withdrawn each day for 12 total days, and assayed at 40°C by the standard esterase assay.
(PDF)

## Abbreviations

PON: human paraoxonase
AHL: acyl-homoserine lactone
TBBL: 5-thiobutilbutyrolactone
*p*NP: *p*-nitrophenol
CE activity: carboxylesterase activity
(3OC12-HSL): *N*-(3-oxododecanoyl)-L-homoserine lactone
m-parathion: methyl-patathion
