# Supplementary figures and images for: An Engineered Version of Human PON2 Opens the Way to Understand the Role of Its Post-Translational Modifications in Modulating Catalytic Activity
Source: PLoS One. 2015 Dec 10;10(12):e0144579. doi: 10.1371/journal.pone.0144579 (PMC4684340; doi:10.1371/journal.pone.0144579)

**S1 Figure. Determination of the optimal temperature for esterase activity.**

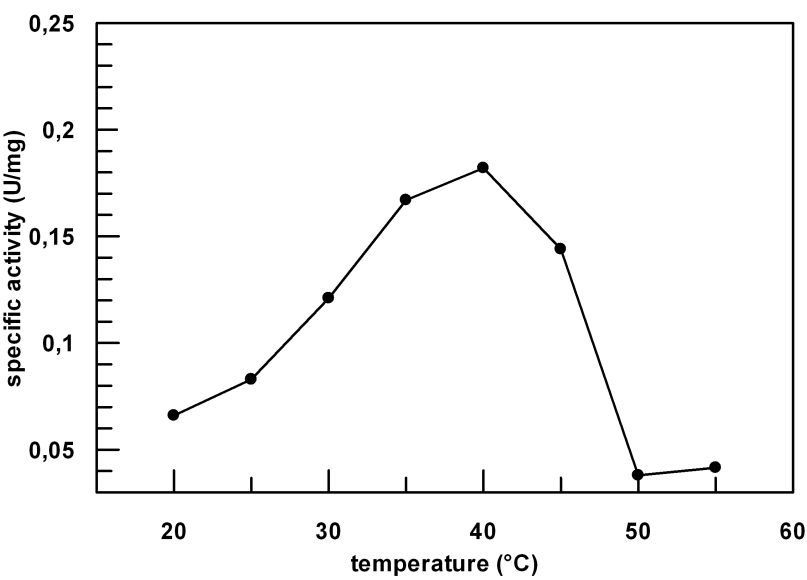

Supplement: S1 Fig — Activity was measured over the range 20–55°C, using the standard esterase assay, pNP-propionate was used as substrate. The dependence of enzymatic activity on temperature was studied over the range of 20–55°C, using pNP-propionate in the standard assay conditions. The optimal temperature was confirmed also for the lactonase activity on TBBL. (PDF) [file pone.0144579.s001.pdf]

**S2 Figure. Determination of the optimal pH for esterase activity.**

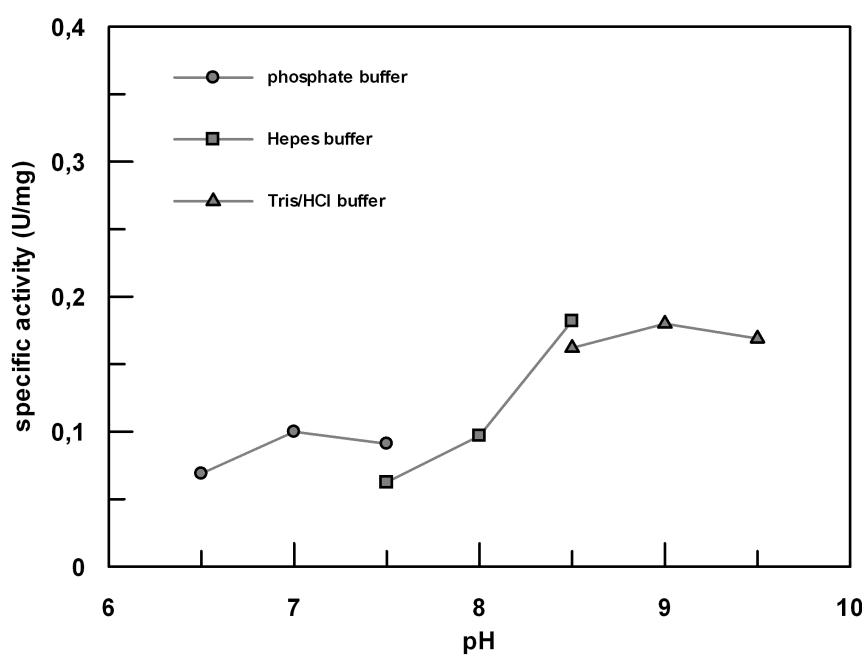

Supplement: S2 Fig — Assays were done at 40°C, using pNP-propionate as substrate. Buffer used were: 20 mM Na2HPO4/NaH2PO4 over the range 6.5–7.5 (circles); 20 mM Tris/HCl over the range 7.5–8.5 (squares); 20 mM Hepes over the range 8.5–9.5 (triangles). The assays were carried out in duplicate or triplicate and the results were the means of two independent experiments. The optimal pH was confirmed also for the lactonase activity on TBBL. (PDF) [file pone.0144579.s002.pdf]

S3 Figure. Thermal stability.

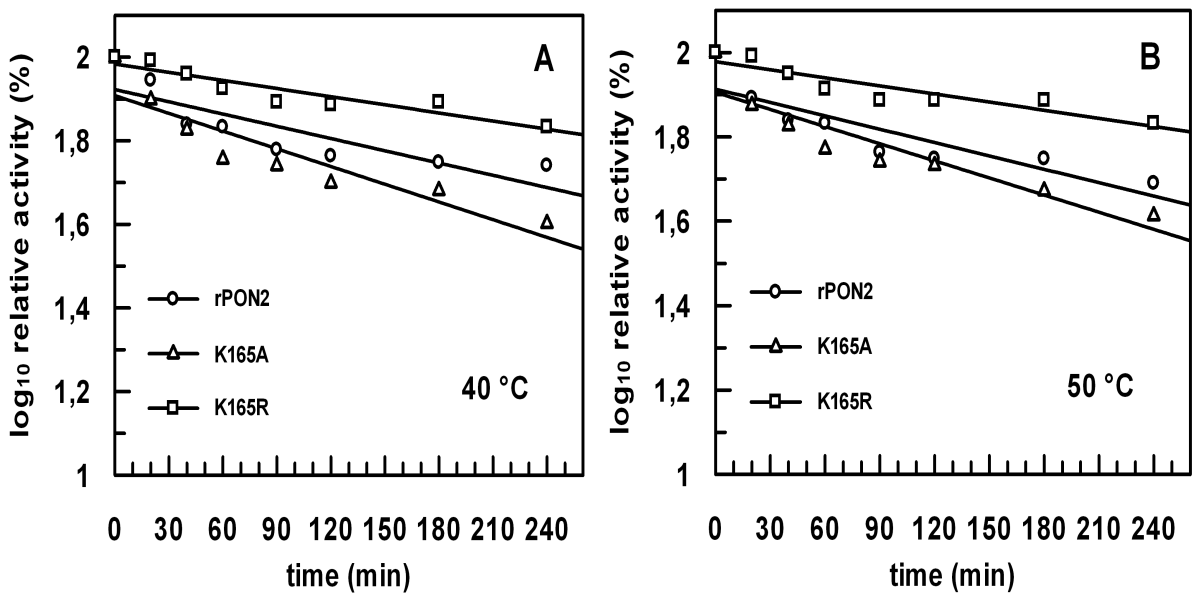

Supplement: S3 Fig — Determinations were made after incubation at 40°C (A) and 50°C (B). The thermal stability of rPON2 and its mutants was assayed at 40 and 50°C. Pure enzymes (0.2 mg/ml in 20 mM Hepes pH 8.5 containing CaCl2 0.5 mM) were incubated in seals glass tubes. Aliquots were withdrawn at time and assayed at 40°C in standard esterase assay. (PDF) [file pone.0144579.s003.pdf]

**S4 Figure. Enzyme stability.**

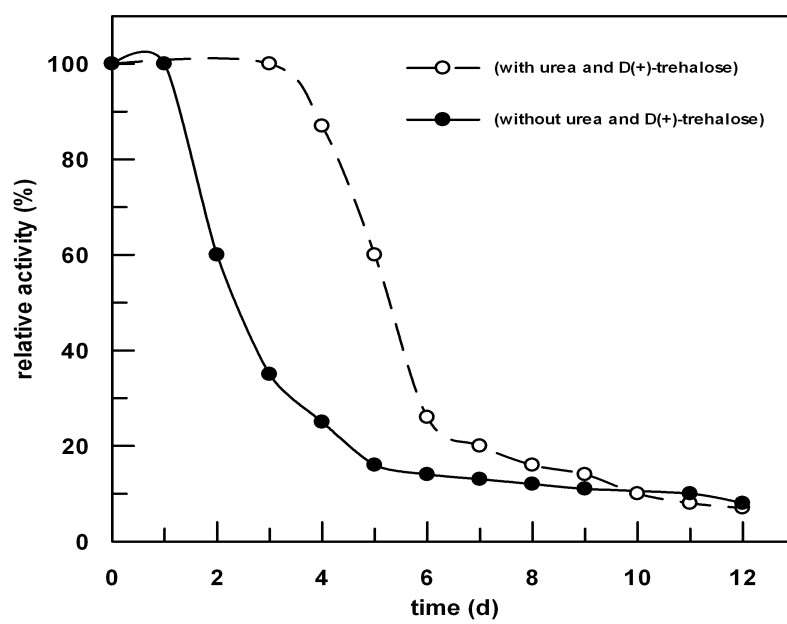

Supplement: S4 Fig — rPON2 at 0.2 mg/ml was dialysed against 20 mM Hepes pH 8.5 containing (open circles) urea 0.25 M and D(+)-trehalose (0.1%) or not (full circles), at 4°C for 12 days. The activity was reported as residual activity respect to the value measured at t0 of incubation. The stability of rPON2 was studied maintaining the enzyme (0.2 mg/ml) at 4°C in storage buffer 20 mM Hepes pH 8.5 containing 0.5 mM CaCl2 /0.25 M urea/ 0.1% (w/v) D(+)-trehalose and in dialysis against storage buffer with or without urea and D(+)-trehalose. Aliquots were withdrawn each day for 12 total days, and assayed at 40°C by the standard esterase assay. (PDF) [file pone.0144579.s004.pdf]
